# Socioeconomic Trends in Palliative Care: A Six-Year Study

**DOI:** 10.7759/cureus.71274

**Published:** 2024-10-11

**Authors:** Andrej Sodoma, Muhammad Wahdan Naseeb, Samuel Greenberg, Nicholas J Knott, Jonathan Arias, Argirios Skulikidis, Mary Makaryus

**Affiliations:** 1 Internal Medicine, South Shore University Hospital, Bay Shore, USA; 2 Anesthesia and Critical Care, Renaissance School of Medicine, Stony Brook University, Stony Brook, USA; 3 College of Osteopathic Medicine, New York Institute of Technology, Jonesboro, USA; 4 Physical Medicine and Rehabilitation, NYC Health and Hospitals, Queens, USA; 5 Palliative Care, South Shore University Hospital, Bay Shore, USA

**Keywords:** geographic variation, healthcare inequalities, hospital size, logistic regression analysis, medicaid, medicare, national inpatient sample, palliative care, socioeconomic disparities

## Abstract

The use of palliative care (PC) is on the rise in the USA, with clear benefits to patients, families, hospitals, and insurance companies. Our study investigates trends in PC utilization, focusing on socioeconomic characteristics. The National Inpatient Sample (NIS) from 2015 to 2020 was used to identify adults hospitalized in the United States (US). International Classification of Diseases, Tenth Revision (ICD-10), was used for PC encounters, code Z51.5. An equal number of random records, stratified by year and without this code, were selected to serve as controls. Records were analyzed for baseline characteristics using a chi-square test. Adjusted odds ratios (ORs) of receiving PC were calculated using multivariate logistic regression. Men were more likely to receive PC consults (OR: 1.07, confidence interval (CI): 1.06-1.08). Medicare/Medicaid holders’ PC access was limited compared to private insurance holders (0.89, 0.86-0.93). Racial minorities, particularly Hispanics (0.9, 0.86-0.95) and Blacks (0.83, 0.77-0.88), were less likely to engage in PC. Compared to urban teaching hospitals, rural hospitals had a decreased rate of PC utilization (0.53, 0.49-0.57). Smaller hospitals had significantly fewer PC referrals than large hospitals (0.80, 0.76-0.85). A lower socioeconomic status was associated with a reduced propensity to utilize PC services compared to an upper socioeconomic status (0.91, 0.87-0.96). Our analysis shows that socioeconomic factors strongly influence PC access. This highlights important inequities that require measures to improve equitable PC access across demographic groups.

## Introduction

According to the World Health Organization (WHO), palliative care (PC) is an approach that improves the quality of life of patients (adults and children) and their families who are facing problems associated with life-limiting illnesses. It prevents and relieves suffering through the early identification, correct assessment, and treatment of pain and other issues, whether physical, psychosocial, or spiritual [1]. PC focuses primarily on anticipating, preventing, diagnosing, and treating symptoms experienced by patients with a serious or life-limiting illness, and helping patients and their families make medically important decisions. The ultimate goal of PC is to improve the patients' and families' quality of life, regardless of diagnosis. For PC, the WHO recommends starting as early as possible during any chronic, ultimately fatal illness [2].

The benefits of PC have now been shown in multiple clinical trials, with increased patient and provider satisfaction, equal or better symptom control, more discernment of and honoring choices about the place of death, fewer and fewer intensive hospital admissions in the last month of life, less anxiety and depression, less caregiver distress, and cost savings [3]. In the past decade, PC has been one of the most rapidly growing healthcare fields in the United States (US) [4,5].

Despite the heightened adoption of PC, substantial disparities persist. There is extensive documentation indicating pronounced racial and ethnic inequities in the access and quality of PC, particularly observed among certain minority groups [6-8]. Nevertheless, there is a dearth of research and attention concerning PC disparities when considering socioeconomic factors. Our investigation delves into the evolving patterns of PC utilization, with a particular emphasis on examining how these trends relate to various socioeconomic characteristics. Our study aims to shed light on the subtle dynamics influencing access to and utilization of PC services by examining the association between PC utilization and socioeconomic determinants.

## Materials and methods

Data source

Our study is a retrospective cohort study using the combined 2015 through 2020 National Inpatient Sample (NIS), an initiative provided by the Healthcare Cost and Utilization Project (HCUP) [9]. The NIS is one of the largest all-payer databases available in the US and is maintained by the Agency for Healthcare Research and Quality (AHRQ) [9]. It comprises over seven million unweighted records and over 35 million weighted hospital encounters annually [9]. The data provided in the database is initially unweighted; then using an algorithm provided by HCUP, it is converted to weighted data, which allows for estimates on the national level [9]. The Institutional Review Board (IRB) approval is not a requirement as the NIS includes de-identified patient information that is publicly available.

 Study population

The NIS includes a 20% random sample of all inpatient hospitalizations from over 45 states and contains one primary diagnosis and up to 39 secondary diagnoses using the International Classification of Diseases, Tenth Revision (ICD-10), and 29 secondary diagnoses with the ICD-9-CM codes [9]. ICD codes were used to identify adults hospitalized in the US who received the ICD-10 code Z51.5: encounter for PC. An equal number of random records, stratified by year and without this code, were selected to serve as controls. Records were analyzed for baseline characteristics: sex (male, female), age (18-39, 40-64, 65-74, 75+), insurance (private versus Medicare/Medicaid), race (White, Black, Hispanic, Asian, Native American, Other), Charlson Comorbidity Index (CCI), region (Northeast, West, Midwest, South), hospital bed size (large, medium, small), hospital status (urban teaching, urban nonteaching, rural), and income status (first, second, third, and fourth income quartile).

 Statistical analysis 

Baseline characteristics were analyzed using a chi-square test. Adjusted odds ratios (ORs) of receiving PC were calculated using multivariate logistic regression. Stata version 17 (StataCorp LLC, College Station, TX) was utilized for all statistical analyses [10].

## Results

This extensive research investigation examined a total of 5,242,014 patients who had PC during admission and an equivalent number of non-PC patients during admission with comparable characteristics. Nationwide trends in the utilization of PC among inpatients from 2015 to 2020 were examined. The analysis revealed an uptrend in the overall number of inpatients receiving PC services during the study period. There was a statistically significant difference between male and female PC patients (1.07, 1.06-1.08), as seen in Table 1. 

**Table 1:** Adjusted odds ratio and confidence intervals for demographics of PC patients in the United States from NIS data, 2015-2020 CCI: Charlson Comorbidity Index; OR: odds ratio; CI: confidence interval; NIS: National Inpatient Sample Adjusted OR and 95% CI. Hospital bed size is determined by the NIS classifications [11]

	2015	2016	2017	2018	2019	2020	Overall
Number of patient visits	674,135	767,484	853,735	908,005	965,555	1,073,100	5,242,014
Sex							
Male	1.00	1.00	1.00	1.00	1.00	1.00	1.00
Female	1.07 (1.05-1.09)	1.07 (1.05-1.09)	1.06 (1.04-1.08)	1.06 (1.04-1.08)	1.07 (1.05-1.08)	1.08 (1.06-1.09)	1.07 (1.06-1.08)
Age							
18-39	1.00	1.00	1.00	1.00	1.00	1.00	1.00
40-64	1.06 (0.99-1.14)	1.10 (1.01-1.19)	1.13 (1.05-1.22)	1.08 (1.01-1.16)	1.17(1.09-1.25)	1.42 (1.33-1.51)	1.17 (1.14-1.21)
65-74	1.09 (1.00-1.17)	1.16 (1.06-1.26)	1.21 (1.12-1.31)	1.21 (1.13-1.30)	1.28 (1.19-1.39)	1.73 (1.61-1.86)	1.30 (1.26-1.34)
75+	2.12 (1.95-2.29)	2.23 (2.04-2.43)	2.37 (2.18-2.57)	2.31 (2.14-2.50)	2.46 (2.27-2.67)	3.29 (3.05-3.55)	2.50 (2.41-2.58)
Insurance							
Private	1.00	1.00	1.00	1.00	1.00	1.00	1.00
Medicare/Medicaid	0.89 (0.86-0.93)	0.90 (0.86-0.94)	0.92 (0.88-0.95)	0.91 (0.88-0.95)	0.92 (0.88-0.96)	0.94 (0.90-0.98)	0.92 (0.90-0.93)
Race							
White	1.00	1.00	1.00	1.00	1.00	1.00	1.00
Black	0.90 (0.86-0.95)	0.94 (0.90-0.98)	0.94 (0.91-0.98)	0.93 (0.90-0.97)	0.94 (0.91-0.97)	0.96 (0.93-1.00)	0.94 (0.92-0.95)
Hispanic	0.83 (0.77-0.88)	0.83 (0.78-0.87)	0.84 (0.80-0.88)	0.85 (0.81-0.89)	0.87 (0.82-0.91)	0.99 (0.95-1.04)	0.87 (0.85-0.89)
Asian	0.99 (0.93-1.06)	1.03 (0.96-1.10)	0.99 (0.93-1.05)	1.06 (1.00-1.13)	1.05 (0.99-1.11)	1.07 (1.01-1.13)	1.03 (1.01-1.06)
Native American	0.97 (0.82-1.15)	1.10 (0.93-1.29)	1.04 (0.90-1.21)	1.02 (0.91-1.15)	0.98 (0.86-1.12)	1.12 (1.00-1.25)	1.04 (0.98-1.10)
Other	0.91 (0.83-1.00)	0.91 (0.84-0.99)	0.91 (0.84-0.98)	0.94 (0.85-1.03)	0.99 (0.91-1.06)	1.06 (0.98-1.14)	0.96 (0.93-0.99)
CCI							
0	1.00	1.00	1.00	1.00	1.00	1.00	1.00
1-2	5.71 (5.28-6.16)	4.87 (4.52-5.24)	4.82 (4.50-5.17)	4.96 (4.64-5.31)	4.83 (4.50-5.17)	4.65 (4.32-5.00)	4.91 (4.77-5.06)
3+	44.18 (40.43-48.28)	33.19 (30.43-36.19)	30.62 (28.33-33.10)	30.44 (28.12-32.95)	27.67 (25.53-29.99)	20.85 (19.14-22.71)	29.21 (28.23-30.24)
Region							
Northeast	1.00	1.00	1.00	1.00	1.00	1.00	1.00
Midwest	1.12 (1.03-1.21)	1.18 (1.09-1.28)	1.22 (1.12-1.32)	1.17 (1.07-1.28)	1.13 (1.04-1.22)	1.12 (1.04-1.21)	1.15 (1.12-1.19)
South	1.09 (1.01-1.18)	1.16 (1.08-1.25)	1.12 (1.04-1.21)	1.09 (1.01-1.19)	1.08 (1.00-1.17)	1.04 (0.96-1.12)	1.09 (1.06-1.13)
West	1.23 (1.13-1.33)	1.25 (1.16-1.35)	1.29 (1.20-1.40)	1.17 (1.07-1.27)	1.14 (1.05-1.24)	1.04 (0.97-1.13)	1.17 (1.13-1.21)
Hospital bed size							
Large	1.00	1.00	1.00	1.00	1.00	1.00	1.00
Medium	0.67 (0.62-0.71)	0.65 (0.61-0.69)	0.65 (0.61-0.69)	0.65 (0.60-0.69)	0.68 (0.64-0.73)	0.71 (0.67-0.75)	0.67 (0.65-0.69)
Small	0.80 (0.76-0.85)	0.82 (0.77-0.87)	0.83 (0.78-0.87)	0.82 (0.78-0.87)	0.85 (0.80-0.90)	0.87 (0.82-0.91)	0.83 (0.82-0.85)
Hospital status							
Urban teaching	1.00	1.00	1.00	1.00	1.00	1.00	1.00
Urban nonteaching	0.70 (0.66-0.74)	0.70 (0.66-0.74)	0.70 (0.66-0.74)	0.71 (0.67-0.75)	0.71 (0.67-0.76)	0.74 (0.70-0.78)	0.72 (0.70-0.73)
Rural	0.53 (0.49-0.57)	0.53 (0.49-0.57)	0.55 (0.50-0.59)	0.55 (0.51-0.60)	0.57 (0.52-0.62)	0.58 (0.53-0.63)	0.55 (0.53-0.57)
Patient income status based on zip code							
1st quartile (richest)	1.00	1.00	1.00	1.00	1.00	1.00	1.00
2nd quartile	0.98 (0.94-1.02)	0.98 (0.94-1.02)	0.96 (0.92-0.99)	0.96 (0.92-0.99)	0.96 (0.92-0.99)	0.97 (0.94-1.01)	0.97 (0.95-0.98)
3rd quartile	0.97 (0.93-1.02)	0.96 (0.92-1.00)	0.96 (0.93-1.01)	0.96 (0.92-1.00)	0.97 (0.93-1.01)	0.99 (0.95-1.03)	0.97 (0.95-0.99)
4th quartile (poorest)	0.91 (0.87-0.96)	0.91 (0.86-0.95)	0.91 (0.87-0.95)	0.92 (0.88-0.96)	0.93 (0.89-0.97)	0.95 (0.91-0.99)	0.93 (0.91-0.94)

**Figure 1:**
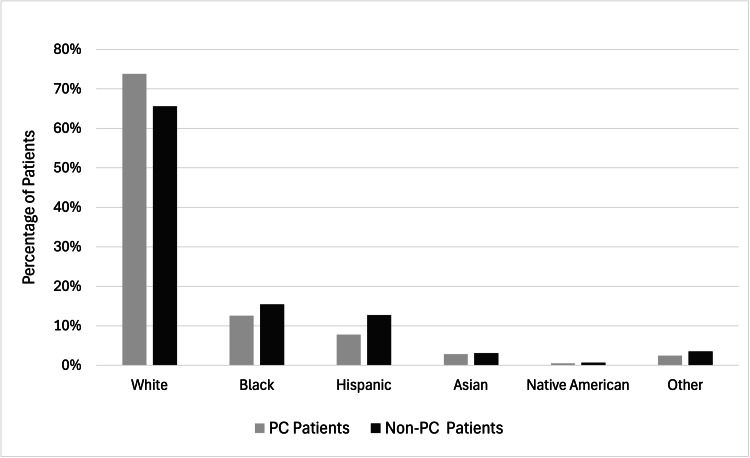
Ethnic differences in patients receiving PC vs. non-PC in the United States using the NIS data, 2015-2020 PC: Palliative care; NIS: National Inpatient Sample Each ethnic group disparity has a p < 0.0001. p < 0.05 is considered significant using the chi-square test

The disparity in ethnic patterns between PC and non-PC populations was found to be statistically significant. PC patients were predominantly White at 73.83%, followed by 12.59% Black, 7.78% Hispanic, 2.85% Asian, 0.50% Native American, and 2.44% "other" (p < 0.0001), as represented in Table 2. Similarly, non-PC patients were largely identified as White at 65.58%, with 15.44% Black, 12.70% Hispanic, 3.10% Asian, 0.64% Native American, and 3.54% classified as "other" (p < 0.0001) (Figure 1). However, disparities were observed based on race, with Black (0.94, 0.92-0.95) and Hispanic (0.87, 0.85-0.89) patients being less likely to receive PC compared to White patients (Table 1). Notably, this discrepancy was not statistically significant in 2020 (Black 0.96, 0.93-1.00; Hispanic 0.99, 0.95-1.04) (Table 1), suggesting potential improvements in equitable access over time.

**Table 2:** Distribution of demographics of PC and non-PC (controls) in the United States from NIS data, 2015-2020 PC: Palliative care: NIS: National Inpatient Sample Hospital bed size is determined by the NIS classifications [11]. p < 0.05 is considered significant using the chi-square test

	Palliative care	Controls	p-value
Weighted N in sample	5,242,014	5,242,014	
Sex			<0.0001
Male	51.92%	56.49%	
Female	48.08%	43.51%	
Age			<0.0001
18-39	3.55%	35.65%	
40-64	21.43%	28.06%	
65-74	21.09%	16.02%	
75+	53.93%	20.28%	
Insurance			
Private	15.44%	29.53%	
Medicare/Medicaid	77.97%	63.23%	
Race			<0.0001
White	73.83%	64.58%	
Black	12.59%	15.44%	
Hispanic	7.78%	12.70%	
Asian	2.85%	3.10%	
Native American	0.50%	0.64%	
Other	2.44%	3.54%	
CCI			<0.0001
0	1.39%	33.15%	
1-2	4.10%	17.78%	
3+	94.52%	49.07%	
Region			<0.0001
Northeast	18.37%	18.68%	
Midwest	23.30%	21.38%	
South	38.68%	40.35%	
West	19.65%	19.59%	
Hospital bed size			<0.0001
Large	54.08%	50.42%	
Medium	28.63%	29.06%	
Small	17.29%	20.52%	
Hospital status			<0.0001
Urban teaching	74.10%	70.19%	
Urban nonteaching	19.52%	21.25%	
Rural	8.56%	6.39%	
Patient income status based on zip code			<0.0001
1st quartile (richest)	22.02%	19.79%	
2nd quartile	24.58%	23.61%	
3rd quartile	25.96%	25.93%	
4th quartile (poorest)	27.44%	30.67%	

Insurance coverage analysis indicated significant discrepancies, with the vast majority of both PC and non-PC patients covered by Medicare/Medicaid (15.44% private, 77.97% Medicare/Medicaid; 29.53% private, 63.23% Medicare/Medicaid) (p < 0.0001), as shown in Table 2. Additionally, patients with private insurance had significantly higher access to PC services compared to those covered by Medicare/Medicaid (0.92, 0.90-0.93) (Table 1). PC patients primarily had a CCI score of 3+ at 94.52%, with 1.39% with CCI of 0 and 4.10% with CCI of 1-2 (p < 0.0001) (Table 2). Non-PC patients exhibited a heterogeneous distribution, with 33.15% CCI of 0, 17.78% CCI of 1-2, and 49.07% CCI of 3+ (p < 0.0001) (Table 2).

**Figure 2:**
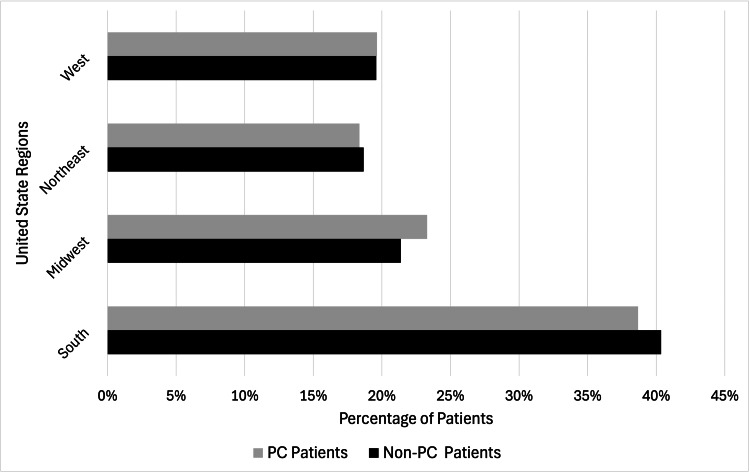
Regional differences in patients receiving PC vs. non-PC in the United States using NIS data, 2015-2020 PC: Palliative care; NIS: National Inpatient Sample Each regional disparity has a p < 0.0001. p < 0.05 is considered significant using the chi-square test

Geographic analysis revealed a substantial regional correlation between the PC and non-PC groups. PC patients were mostly concentrated in the South (38.68%), followed by the Midwest (23.30%), Northeast (18.37%), and West (19.65%) (p < 0.0001) (Table 2). Non-PC patients were primarily distributed in the South (40.35%), followed by the Midwest (21.38%), Northeast (18.68%), and West (19.59%) (p < 0.0001) (Figure 2). The geographic analysis revealed significant disparities in access to PC services across different regions of the US. In comparison to the Northeast region, the Midwest (1.15, 1.12-1.19), South (1.09, 1.06-1.13), and West (1.17, 1.13-1.21) regions exhibited notably higher accessibility to PC services, as represented in Table 1.

Large hospitals (54.08%) had the highest proportion of PC patients, followed by medium-sized (28.63%) and small hospitals (17.29%) (Table 2). Larger hospitals demonstrated greater access to PC services compared to small (0.83, 0.82-0.85) or medium-sized (0.67, 0.65-0.69) hospitals (Table 1). Moreover, patients in urban teaching hospitals (75.10%) were significantly more likely to receive PC compared to those in urban nonteaching hospitals (19.52%) and rural hospitals (8.56%) (Table 2).

PC patients' income was distributed among quartiles (22.02%, 24.58%, 25.96%, and 27.44%, respectively) (p < 0.0001) (Tabel 2). Furthermore, patients in the lowest quartile of financial status were significantly less likely to receive PC services (0.93, 0.91-0.94) (Table 1), underscoring socioeconomic disparities in access to PC and the need for targeted interventions to address these inequities.

## Discussion

PC represents a vital aspect of modern healthcare, providing specialized support to patients with serious illnesses to enhance their quality of life. However, while the core aim of PC remains consistent, its utilization and distribution across different demographic groups can vary significantly. The six-year study presented here delves deep into the socioeconomic trends characterizing PC admissions, shedding light on disparities that warrant attention within the healthcare landscape.

One of the prominent findings of this study is the notable gender discrepancy among PC recipients. The analysis indicates a higher proportion of male patients receiving PC than females, contrasting with the gender distribution in non-PC admissions. This suggests potential variations in healthcare-seeking behaviors or disease prevalence between genders, which warrants further investigation to tailor interventions accordingly. A scoping review conducted in 2022 also reported significant gender disparity in the different domains of PC, including care context, caregiving, symptom experience, care preferences, living situation, and coping strategies [12]. Age distribution disparities were also evident, with PC patients being significantly older on average compared to non-PC patients. This aligns with the existing literature indicating that older individuals are more likely to require PC due to the higher prevalence of chronic and life-limiting illnesses in this demographic [13]. However, it also underscores the importance of proactive care planning and advance directives for elderly populations to ensure that their preferences are respected and appropriate care is provided.

Ethnicity and insurance coverage emerge as significant factors influencing access to PC. Ethnic patterns revealed a predominance of White patients in both PC and non-PC cohorts, highlighting potential disparities in access to PC services among minority groups. A cohort in 2020 compared the differences in end-of-life care between Black and White patients and found that Black patients were less likely to use PC despite the increase in hospice care in recent years [14]. Moreover, the overwhelming reliance on Medicare/Medicaid for both palliative and non-PC underscores the critical role of public payers and government-sponsored healthcare programs in facilitating access to end-of-life care [15]. Addressing cultural barriers, increasing cultural competency among healthcare providers, and enhancing outreach efforts within underserved communities are essential steps to promote equitable access to PC across diverse populations. Healthcare financing mechanisms play a critical role in shaping access to PC services and the need for policy interventions to ensure adequate reimbursement for PC delivery.

Geographic variations in PC utilization underscore the influence of regional healthcare dynamics on service delivery. The concentration of PC admissions in the Southern and Midwestern regions suggests potential disparities in healthcare access and resource allocation. Understanding regional differences is crucial for developing targeted interventions to address disparities and enhance equitable access to PC services nationwide. A population-based study examining PC provision in the US identified an increase in PC utilization in the last 15 years, but a significant geographic variation was found across the different states of the US [16]. By identifying regions with lower utilization rates, healthcare policymakers can implement strategies to increase awareness, improve infrastructure, and expand PC delivery networks in underserved areas. Additionally, the distribution of PC patients across different hospital sizes highlights the importance of healthcare infrastructure in facilitating access to specialized services. 

Lastly, the income distribution analysis revealed nuanced differences between PC and non-PC patients, emphasizing the intersectionality of socioeconomic factors in healthcare utilization. While income distribution among PC patients was found to be relatively uniform across quartiles, non-PC admissions exhibit a skewed distribution toward higher-income quartiles. To mitigate socioeconomic disparities in PC access, systemic changes such as expanding insurance coverage, increasing funding for safety net programs, and implementing reimbursement models that incentivize PC provision in underserved communities are essential. Previous literature has compared the impact of socioeconomic status on the utilization of PC. Davies et al. and Mason et al. synthesized evidence across multiple high-income countries, revealing low socioeconomic position as a risk factor for suboptimal end-of-life care outcomes, similar to the findings of our study [17,18]. In contrast to the findings reported by Vestergaard et al. [19], which indicated limited socioeconomic disparities in the utilization of healthcare services at the end of life among both cancer and noncancer patients in Denmark, our study revealed significant demographic and socioeconomic differences within the PC population in the US. While Vestergaard et al. found no clear temporal trends in the use of various healthcare services among patients with different socioeconomic positions, we identified pronounced disparities in gender distribution, age demographics, ethnic patterns, insurance coverage, comorbidity burden, geographic distribution, and financial status between patients admitted under PC and those in non-PC settings. 

To mitigate the socioeconomic gap in care provision, resource allocation strategies must be informed by a comprehensive understanding of the underlying determinants of healthcare disparities [17]. Targeted investments in PC infrastructure, workforce development, and community-based programs are essential to expand access to PC services in underserved areas. This may involve establishing PC teams in rural and urban healthcare facilities, enhancing training programs to increase cultural competency among healthcare providers, and implementing outreach initiatives to raise awareness about PC among marginalized communities. Additionally, reallocating resources toward preventive care, early screening, and chronic disease management can help reduce the need for PC interventions among disadvantaged populations [20]. By addressing upstream determinants of health and promoting equitable access to primary care services, healthcare systems can mitigate disparities in PC utilization and improve overall health outcomes.

Addressing socioeconomic disparities in PC provision also requires systemic changes within the healthcare system. Policy reforms aimed at expanding insurance coverage, eliminating financial barriers to care, and promoting value-based payment models can facilitate equitable access to PC services for all patients, regardless of socioeconomic status [21]. Furthermore, efforts to integrate PC into existing healthcare delivery models, such as primary care practices and specialty clinics, can enhance accessibility and ensure timely access to PC services throughout the continuum of illness. Moreover, fostering collaboration between healthcare stakeholders, community organizations, and policymakers is essential to develop and implement evidence-based interventions that address the social determinants of health and promote health equity. By adopting a multidimensional approach that addresses both clinical and social determinants of health, healthcare systems can effectively lessen the socioeconomic gap in PC provision and advance health equity for all patients.

Limitations

While this study does highlight several discrepancies, various limitations arise. First, the scope of this research only pertains to a six-year period, which needs to capture how these practices have trended in the past; further studies should be done to document the time before 2015 and after 2020. A broader timeline may demonstrate exciting changes in the PC landscape and how discrepancies have changed. Second, while there are discrepancies among patients who do receive PC, the NIS dataset does not have data on the quality of care received. Also, due to limitations of the NIS dataset, one can only determine if PC was in the plan rather than when PC became part of the treatment plan. Lastly, while there are socioeconomic differences, this study did not look at the cultural beliefs that may influence one's decision on palliative medicine. Addressing these limitations in future research may provide insight into narrowing the discrepancies and providing optimal palliative medicine.

## Conclusions

In conclusion, our findings highlight the multifaceted nature of socioeconomic disparities in PC utilization and underscore the need for comprehensive strategies to promote equitable access to PC services. By addressing these disparities through targeted interventions, policy reforms, and resource allocation, we can ensure that all patients receive high-quality, person-centered PC that aligns with their preferences and values, regardless of socioeconomic status or demographic characteristics.
